# A report on the global conference on research and application of Chinese herbal medicine-derived extracellular vesicle-like particles (2024): utilizing medicinal plant-derived extracellular vesicles to advance global pharmaceutical development

**DOI:** 10.20517/evcna.2024.98

**Published:** 2025-01-18

**Authors:** Yingqi Cao, Zhengting Wu, Huijing Li, Jiawen Shen, Dongxiao Li, Tianxin Qiu, Zimei Chen, Qing Zhao, Kewei Zhao

**Affiliations:** ^1^The Third Clinical Medical College of Guangzhou University of Chinese Medicine, Guangzhou 510375, Guangdong, China.; ^2^Guangdong Engineering Research Center of Chinese Herbal Vesicles, Guangzhou University of Chinese Medicine, Guangzhou 510375, Guangdong, China.; ^3^Guangzhou Key Laboratory of Chinese Medicine Research on Prevention and Treatment of Osteoporosis, The Third Affiliated Hospital of Guangzhou University of Chinese Medicine, Guangzhou 510378, Guangdong, China.; ^#^Authors contributed equally.

## INTRODUCTION

The 2024 Global Conference on Research and Application of Chinese herbal medicine-derived extracellular vesicle-like particles (CHM-EVLPs) was hosted in Guangdong on August 24-25, 2024. The organizers of this conference are the Guangzhou University of Chinese Medicine and the Guangdong Provincial Association of Chinese Medicine, while the Third Affiliated Hospital of Guangzhou University of Chinese Medicine serves as the co-organizer. Professor Kewei Zhao from the Guangdong Engineering Research Center for Chinese Herbal Vesicles chaired this conference and was the event’s main organizer. This periodic meeting in Guangdong intended to actively involve and bring into focus CHM-EVLP experts and scientists at the early career stage, driving interactions and promoting collaborations. The conference focused on extracellular vesicles (EVs) derived from plants, fruits, and vegetables, emphasizing fundamental research, extraction techniques, quality evaluation, interdisciplinary studies, disease treatment, and clinical applications of CHM-EVLPs. Furthermore, the conference delved into the opportunities and challenges associated with CHM-EVLPs in both traditional medicine and modern innovation. The conference was held in a hybrid format, combining online and offline participation.

The conference drew over 350 participants and received support from a range of sponsors, including the Guangzhou University of Chinese Medicine, the Guangdong Provincial Association of Chinese Medicine, the Guangdong Engineering Research Center for Chinese Herbal Vesicles, the Chinese Society of Extracellular Vesicles, the Chinese Research Hospital Association, the Specialized Committee on Chinese Herbal Medicine-derived Extracellular Vesicles of the Guangdong Association of Chinese Medicine, and the Third Affiliated Hospital of the Guangzhou University of Chinese Medicine. All participants had an in-depth overview of technical requirements and advances in isolation, characterization, basic research, and potential clinical application of EVs. The program featured three keynote lectures by internationally renowned EVs scientists, along with thirty-five presentations by young scientists selected from submitted abstracts. Furthermore, experts from diverse disciplines were convened for a roundtable discussion on four key topics.

At the conference, all attendees showed a natural inclination toward interpersonal interaction, thus creating an enjoyable atmosphere. Thanks to all the participants, the organizing team, and the supporting staff, the meeting provided an excellent platform for productive discussions. We report on the experts’ lectures and summarize the main scientific content below.

## KEYNOTE LECTURES

**Academician Liu Liang** (Guangzhou University of Chinese Medicine, State Key Laboratory of Quality Research in Chinese Medicine, Macau University of Science and Technology) delivered a keynote speech on fostering the development of new quality productive forces and creating scientific and technological innovation in traditional Chinese medicine (TCM). Academician Liu emphasized that CHM-EVLPs represent a new field and breakthrough in TCM research, potentially becoming a key factor in promoting the modernization and industrialization of Chinese medicine. Meanwhile, establishing the CHM-EVLP pilot platform in Guangdong marks a significant step forward in the research and application of CHM-EVLPs. Additionally, Academician Liu highlighted the innovative potential of CHM-EVLPs in drug development, especially in extraction standardization, automation, and the establishment of a quality evaluation system. He called for enhanced international cooperation to advance the modernization and internationalization of TCM. Academician Liu’s speech painted a grand blueprint for CHM-EVLP research and guided future work directions.

**Professor Qian Wang** (Chinese Society of extracellular vesicles) presented the core idea of “Small Vesicles, the Great World” and explored the research and application prospects of CHM-EVLPs in the field of TCM. He pointed out that EVs carry a new field and breakthrough in the research of TCM, potentially becoming a key factor in promoting the modernization and industrialization of Chinese medicine. Moreover, the establishment of the CHM-EVLP pilot platform in Guangdong Province demonstrates the potential of CHM-EVLPs in the modernization of TCM. Professor Wang further discussed the innovative potential of CHM-EVLPs in disease treatment and drug delivery, especially in targeted precision therapy and drug carrier delivery. He called for strengthened academic exchanges and domestic and international cooperation to promote the modernization and internationalization of TCM, allowing CHM-EVLPs to have a greater impact on global health.

**Professor Chunguang Li** (Western Sydney University, Australia) cited that the internationalization of TCM is a major trend, and the recognition and application of TCM globally are rising. He also referred to the consensus of the CHM-EVLP specialists and the considerations that need to be taken into the standardization process, such as nomenclature, characterization, source of materials, and methods of extraction and isolation. He suggested the feasibility of the development of an international standard for CHM-EVLPs. In addition, Professor Li suggested the feasibility of developing international standards for CHM-EVLPs and pointed out the challenges of standardization, including the lack of clear biomarkers, the diversity of material sources, and safety and efficacy issues. He suggested that CHM-EVLPs may be a new direction for TCM research and application and emphasized the crucial role of standardization in advancing this field. His presentation provided a comprehensive overview of the internationalization and standardization issues of CHM-EVLPs.

## PART 1 ADVANCED RESEARCH ON PLANT VESICLES

**Professor Liwen Jiang** (School of Life Sciences, The Chinese University of Hong Kong) provided an in-depth lecture on cross-border RNA communication between plants and eubacteria via EVs, thus affecting gene expression and function. Professor Jiang first outlined the differences between plant-derived vesicles (PDVs) and animal EVs and delved into the role of PDVs in intercellular communication. He discussed his group’s latest findings on the investigation of PDVs, including their transport mechanisms, their role in plant-fungus interactions, and their functions in plant growth and development. Professor Jiang also discussed the potential of PDVs for applications in agriculture and medicine, especially in precision agriculture and novel drug delivery systems.

**Professor Carine de Marcos Lousa** (Centre for Medical Biomedicine, Leeds Beckett University, UK) explored the potential applications of PDVs in biomedicine, including the roles of PDVs in intercellular communication, cell barrier reconstruction, defense mechanisms, and drug delivery, and discussed the similarities and differences in the research between PDVs and mammalian EVs. In addition, Carine shared her colleagues’ research on the extraction, characterization, biogenesis, and secretion of PDVs, and how these PDVs are delivered for transport in living organisms and used for disease treatment.

**Professor Huangge Zhang** (University of Louisville, USA) posited that the capability of PDVs to target tumors or other inflammatory sites specifically is crucial for restoring tissue homeostasis, modulating inflammation, enhancing therapeutic efficacy, and minimizing adverse effects. Professor Zhang elaborated on the isolation techniques, functional characteristics, and therapeutic potentials of PDVs, illustrating their systemic distribution within the body and their impact on particular cell types. His research underscores the anti-inflammatory and antioxidative attributes of PDVs, and their promising role in treating hepatic and gastrointestinal disorders.

**Professor Wei Heng Chng** (Department of Pharmacy, National University of Singapore) addressed the role of PDVs in intercellular communication and their potential in drug delivery. His group investigated how PDVs can be extracted and how specific technological solutions can improve their production and quality. They also researched the physicochemical properties of PDVs. In addition, they worked on the distribution of PDVs *in vivo* and the efficiency of drug delivery, especially in colorectal cancer treatment.

**Professor Wenrong Xu** (Frontier Institute of Medical Sciences, Jiangsu University) and his colleagues investigated a variety of PDVs, including those from aloe vera, onion, and lemon, and their roles in anti-inflammatory, antioxidant, anti-aging, antitumor, and metabolic regulation activities. These PDVs are expected to play significant roles in tissue repair and disease treatment. However, the research and application of PDVs also face several challenges. In terms of purification, the complex structure of plant cells makes it difficult to obtain high-purity vesicles; regarding quality control, the lack of unified standards and detection methods compromises research reproducibility and product stability; large-scale production is constrained by current technological and equipment limitations, hindering widespread application. Furthermore, Professor Xu highlighted the potential of PDVs in precision medicine. The natural components and diversity of PDVs enable personalized treatments, precise targeting of lesion sites, and enhanced therapeutic effects, offering new insights for the advancement of precision medicine.

**Professor David Greening** (Baker Heart and Diabetes Institute, Australia) shared insights into the power of mass spectrometry to decipher EVs from a technological perspective. He presented development in addressing challenges in the fields of proteomics, multi-omics (data integration), and EV/cell signaling that uses biochemical design for groundbreaking discoveries in their protein/lipid architecture, membrane surface, and ability to act as systemic signaling regulators. Such findings in exploring the heterogeneity of EVs, including new EV types, in the context of mammalian systems provided important insights into establishing standardization guidelines, data sharing, and applications for the CHM-EVLP field.

**Professor Ruixi Li** (Southern University of Science and Technology) reviewed basic research on endosomal transport pathways in plant cells, especially the endosomal transport pathways in plant cells, including the synthetic secretory and endosomal degradation pathways. He elaborated on the extraction and function of PDVs and suggested the importance of the plant trans-Golgi in plant endosomal membrane transport. Professor Li also discussed the role of PDVs in forming cell walls and disease resistance and the unique transport pathway of vesicles in plant cells. His research emphasizes the complexity of the endosomal transport pathways in plant cells and their importance in plant physiological processes.

## PART 2 STUDIES ON CHINESE HERBS VESICLES

**Professor Chengyu Jiang** (Chinese Academy of Medical Sciences, China) described the exosome-like nanoparticles (desmosome) in herbal soup, which include small RNAs, lipids, small molecules, trace elements, and proteins, and proposed that desmosomes can be orally consumed to achieve cross-species entry into the cell and *in vivo* for targeting and regulating the expression of disease-related genes. She also introduced a new paradigm of drug discovery based on desmosomes, including the development of herbal mini-nucleic acid drugs against known disease-targeting genes and the identification of effective nucleic acid drugs based on herbs to treat diseases. Professor Jiang’s lecture emphasized the great significance of the modernization of Chinese medicine and suggested the similarities between Chinese and Western medicines in terms of composition and targets, which provides a new perspective for scientific research and international recognition of Chinese medicines.

**Professor Peng Cao** (Nanjing University of Traditional Chinese Medicine) elucidated the role of fresh ginseng EVLP in tumor immunomodulation. Professor Cao compared the preparation process of fresh ginseng EVLP, the analysis of its composition and the mechanism of action in tumor microenvironment. He also referred to the potential application of fresh ginseng EVLP in tumor vaccines and peptide vaccine delivery vehicles, demonstrating the multifaceted applications of fresh ginseng EVLP in tumor therapy. The research of Professor Cao provided a scientific rationale for the application of fresh Chinese medicine in modern medicine and explored its new uses in tumor immunotherapy.

**Professor Hongbo Chen** (School of Pharmaceutical Sciences, Sun Yat-sen University) introduced the inhibitory effect of *Citrus grandis Tomentosa* EVLP on ischemia-reperfusion injury and innate immune response in heart transplantation. They found that *Citrus grandis Tomentosa* EVLP protects cardiomyocytes, reduces the production of reactive oxygen species, and inhibits the inflammatory response of macrophages. They also investigated the potential of *Citrus grandis Tomentosa* EVLP as a drug carrier, especially its targeting and drug delivery efficiency in heart transplantation. Professor Chen also discussed how to enhance the targeting and drug loading of PDVs through genetic engineering modifications and improve the tissue targeting of EVLP in cardiac transplantation through click chemistry techniques.

**Professor Kewei Zhao (**The Third Affiliated Hospital of Guangzhou University of Chinese Medicine) presented the CHM-EVLP theory of channel tropism and its application from basic research to clinical practice, emphasizing the integration of CHM-EVLPs with traditional theories of TCM, especially the association with the theory of “channel tropism”. Meanwhile, Professor Zhao discussed the potential of CHM-EVLPs in clinical applications and how to promote its scientific research and clinical translation, including targeting different CHM-EVLPs to specific organs and their potential in the treatment of osteoporosis, anti-inflammatory and antiviral diseases, etc. Furthermore, Professor Zhao also suggested that CHM-EVLPs can effectively treat osteoporosis and anti-inflammatory and antiviral diseases. Additionally, Professor Zhao proposed CHM-EVLP as a new pharmacodynamic substance to become a new dosage form of TCM and called for more experts and scholars to join together in the research and standardization of CHM-EVLPs.

**Professor Peng Li** (Affiliated Hospital of Guangdong Medical University) elaborated on the potential utility of *Pueraria lobata* EVLP in osteoporosis treatment. They successfully extracted *Pueraria lobata* EVLP by high-speed centrifugation and analyzed its morphology and dimensions in detail using transmission electron microscopy and a nanoparticle size analyzer. The findings demonstrated that *Pueraria lobata* EVLP could effectively promote osteogenic differentiation of bone marrow mesenchymal stem cells and significantly delay the progression of osteoporosis in animal models. In addition, Professor Li also investigated the effect of *Pueraria lobata* EVLP on intestinal flora, revealing its novel mechanism to improve osteoporosis symptoms by regulating the balance of intestinal flora.

**Professor Qing Zhao** (The Third Affiliated Hospital of Guangzhou University of Chinese Medicine) presented nanovesicles derived from *rhizoma drynariae*, which target the ERα signaling pathway to enhance osteogenic differentiation of human bone marrow mesenchymal stem cells and reverse osteoporosis. Her team conducted a comprehensive physical and chemical characterization of the nanovesicles derived from *rhizoma drynariae* and evaluated their biological functions in both *in vitro* and *in vivo* models. Professor Zhao’s research revealed that these EVLP can be absorbed by bone marrow mesenchymal stem cells while promoting their osteogenic differentiation, indicating significant potential for treating osteoporosis.

**Professor Yue Cao** (Medical College of Shaoguan University) investigated the efficacy of EVLP derived from Himalayan Teasel Root in treating osteoporosis. By optimizing the traditional centrifugation method, she successfully isolated highly pure EVLP derived from Himalayan Teasel Root and validated its effectiveness at both cellular and animal levels. The study revealed that these EVLPs can effectively modulate the balance between osteoblasts and osteoclasts, enhance bone density and quality, and mitigate fracture risk. Professor Cao’s research suggests that continuous discontinuous EVLP holds significant potential as an innovative therapeutic intervention.

## PART 3: FUNDAMENTAL AND CUTTING-EDGE RESEARCH ON INTER-SPECIES RNA COMMUNICATION ASSOCIATED WITH CHM-EVLPS

**Professor Qiang Cai** (College of Life Sciences at Wuhan University) elucidated the emerging role of EVs in mediating plant-pathogen interactions, particularly in the context of plant immune responses. His research team unveiled a novel mechanism whereby small RNAs are transferred via EVs to regulate plant immunity and investigated the intricate process of information exchange between plants and pathogens mediated by EVs. This groundbreaking research offers a fresh perspective on understanding how plants fortify themselves through EV-mediated mechanisms.

**Professor Xi Chen** (Nanjing University School of Life Sciences) initially elucidated the evolution of the concept of active constituents within TCM. Traditionally, it was postulated that the active constituents of Chinese medicine primarily comprised small molecular compounds such as alkaloids, copper, and sterols. However, contemporary research has revealed that macromolecules, including polysaccharides, proteins, and nucleic acids, may also serve as efficacious components of Chinese medicine. Professor Chen’s team unveiled the capacity for plant-derived miRNA to be assimilated by the human body via specific pathways and elicit biological functions intrinsically. Their investigation into miRNA within honeysuckle decoction demonstrated that miRNA-2911 can endure in extracellular environments while exhibiting antiviral effects in animal models. Furthermore, they probed into the absorption mechanism of miRNA within honeysuckle decoction and posited its potential uptake through gastric means. Professor Chen’s discourse accentuates both the prospective pharmaceutical value and mechanistic underpinnings of macromolecular RNA within Chinese medicine.

**Professor Yuan Liu** (UT MD Anderson Cancer Center) presented her research on enhancing human disease treatment using PDVs. Her team focuses on therapeutic applications of small RNA and has extensively investigated various delivery systems, ultimately prioritizing the study of exosomes. They have discovered that PDVs possess desirable characteristics such as low immunogenicity, high yield, and cost-effectiveness. The team further explored effective extraction methods for PDVs and optimized their loading capacity with small RNA to enhance treatment efficacy. Moreover, Professor Liu discussed the potential of PDVs in tumor therapy, including targeted delivery of small RNA to tumor cells and evaluation of their effects in animal models.

**Professor Anil K. Sood** (UT MD Anderson Cancer Center) presented the developmental process and current research progress of RNA interference (RNAi) therapy. He highlighted the challenges encountered in the clinical translation of RNAi therapy, including issues related to instability, rapid degradation, and metabolic concerns associated with *in vivo* administration. Additionally, he introduced the utilization of nanoliposome technology in RNAi delivery systems and demonstrated its distribution and application within tumor microenvironments. Professor Sood’s presentation emphasized the potential and future directions of integrating RNAi with nanoliposome technology for disease treatment.

**Professor Hailing Jin** (California State University) investigated the role of PDVs in mediating inter-kingdom RNAi to combat fungal pathogens. Professor Jin’s research team is dedicated to elucidating plant defense mechanisms against fungal infections and has uncovered a natural communication mechanism involving RNA between plants and fungi, termed “inter-kingdom RNA warfare”. Their findings suggest that plants can deliver small RNAs into fungal cells through EVs, thereby suppressing the expression of fungal genes. This not only enhances our comprehension of plant-fungi interactions but also establishes a scientific foundation for developing more efficacious strategies for plant disease management.

### Part 4: CHM-EVLP intersects with materials science

**Professor Bo Xiao** (School of Medicine UESTC) presented the utilization of PDVs in oral nano drug delivery systems, specifically focusing on their application in the treatment of colon diseases. Professor Xiao’s research team has successfully developed an innovative oral nano drug delivery system based on PDVs to enhance therapeutic efficacy for colon diseases. Their findings demonstrate that PDVs exhibit remarkable gastrointestinal stability and targeted uptake by specific immune cells or tumor cells. Furthermore, Professor Xiao discussed the potential applications of PDVs in managing Ulcerative colitis (UC) and colorectal cancer through approaches such as fecal microbiota transplantation and oral medication for modulating gut microbiota.

**Professor Lihua Peng** (The Pharmaceutical Sciences of Zhejiang University) provided a comprehensive overview of recent advancements in diabetic ulcer treatment. The research team employed biomimetic electric field technology to expedite wound healing and ameliorate endothelial cell dysfunction, resulting in the development of a multifunctional integrated dressing. This innovative dressing combines intelligent electroactive hydrogel with actively targeted exosomes, enabling multi-factor regulation and multidimensional treatment within the intricate microenvironment of diabetic ulcers. Through rigorous *in vivo* experiments, it has been empirically demonstrated that this dressing effectively stimulates epithelial regeneration and granulation tissue formation by activating PPI pathways, thereby facilitating collagen remodeling, angiogenesis, and inflammation immune regulation for successful granulation tissue regeneration and epithelialization.

**Professor Junbing Fan** (Basic Medical College of Southern Medical University) presented the extraction technique of EVLPs from fruits and their application in constructing advanced drug delivery systems. Professor Fan’s research team is dedicated to developing biomimetic materials, specifically EVLPs derived from fruits, for the treatment of tumors, particularly gliomas. They conducted comprehensive investigations on the impact of EVLP size, morphology, and surface modification on cellular uptake and drug delivery efficiency. Additionally, Professor Fan discussed strategies to enhance the permeability and drug delivery efficacy of EVLPs through adaptive deformation and multi-pathway endocytosis. This study offers valuable insights into designing innovative drug carriers with improved capacity to overcome physiological barriers.

**Professor Jianhui Rong** (The School of Chinese Medicine, the University of Hong Kong) presented the pathological mechanism and treatment strategies for myocardial infarction, elucidating the potential of puerarin in its therapeutic application. His research team has successfully developed a cardiac-targeted delivery system utilizing nanoparticle technology to enhance the bioavailability and efficacy of puerarin. Furthermore, he discussed the molecular mechanisms underlying puerarin’s cardioprotective effects and highlighted the importance of heart-specific targeting to optimize treatment outcomes.

## PART 5: BASIC AND CUTTING-EDGE RESEARCH ON EVLPS DERIVED FROM FRUITS AND VEGETABLES

**Professor Suhua Qi** (Xuzhou Medical University) presented the potential application of bitter melon EVLP in the treatment of cardiovascular and cerebrovascular diseases, with a particular focus on their protective effects against stroke and heart injury. The bitter melon EVLP was obtained through techniques such as ultra-high-speed centrifugation, followed by a comprehensive analysis of their constituents. The investigation revealed that bitter melon EVLP possesses the ability to traverse the blood-brain barrier, thereby safeguarding against stroke-induced damage by inhibiting iron-mediated cell death pathways and exerting neuroprotective effects. Furthermore, bitter melon EVLP demonstrated efficacy in reducing myocardial cell apoptosis, enhancing mitochondrial structure, and protecting against heart injury. Professor Qi also highlighted additional potential applications of bitter melon EVLP in regulating blood glucose levels, exhibiting antitumor activities, and exerting anti-inflammatory effects while emphasizing their safety profile and stability.

**Professor Weiliang Xia** (Shanghai Jiao Tong University) presented the characterization of EVLP derived from celery and its application in tumor treatment. His team made a novel discovery that celery EVLP contains unique lipid components, which can be efficiently internalized by diverse cell types. Celery EVLP exhibits potent inhibitory effects on tumor cells and holds promise as a drug carrier to enhance the delivery efficiency of chemotherapy agents. Furthermore, celery EVLP demonstrates the ability to downregulate PD-L1 expression and synergize with immune checkpoint inhibitors. Professor Xia also discussed the stability and storage conditions of celery EVLP, along with their potential for clinical translation.

**Professor Wei Li** (School of Laboratory Medicine, Wenzhou Medical College) presented the preparation method, component analysis, and effects of EVLP derived from Sargassum fusiforme. The research team led by Professor Li discovered that Sargassum fusiforme EVLP is abundant in ceramides and possesses potent anti-inflammatory and antioxidant properties. Furthermore, Sargassum fusiforme EVLP exhibited inhibitory effects on inflammatory factor expression and demonstrated protective efficacy against both acute and chronic inflammation in animal models. Additionally, Professor Li explored the potential application of Sargassum fusiforme EVLP in osteoporosis treatment while proposing a scalable production technique for vesicles through hollow fiber ultrafiltration.

**Professor Qi Lv** (Nanjing University of Chinese Medicine) presented the potential application of greengage EVLP in the treatment of UC. The research conducted by Professor Lv’s team revealed that greengage EVLP bubbles possess inhibitory effects on NLRP3 inflammasome activation, thereby effectively alleviating UC symptoms. Furthermore, they investigated the *in vivo* distribution and targeting ability of greengage EVLP toward macrophages, as well as their role in modulating immune responses to improve UC outcomes. This study by Professor Lv provides valuable insights for the development of novel therapeutic approaches for UC.

## PART 6: CHM-EVLP AND INSPECTIONS

**Professor Tong Wang** (College of Life Science and Technology, Jinan University) primarily discussed the role of EVs in major infectious diseases, particularly human immunodeficiency virus (HIV). He provided a comprehensive explanation of the HIV virus life cycle and current intervention strategies. It was highlighted that despite controllable viral load, HIV patients still encounter numerous complications. Professor Wang emphasized how HIV exploits the host cell’s vesicular system to facilitate its life cycle and mentioned the potential application of TCM in treating HIV, specifically highlighting CHM-EVLP’s ability to target macrophages and inhibit inflammation. Additionally, he addressed advancements in HIV DNA detection kit development and their utility for evaluating treatment effectiveness and predicting immune reconstitution deficiency by testing storage banks.

**Professor Kewei Zhao** (The Third Affiliated Hospital of Guangzhou University of Chinese Medicine) presented his insights on the integration of CHM-EVLPs and laboratory medicine, proposing a novel concept for monitoring blood drug concentration in TCM. He explored the significance of blood drug concentration in assessing drug efficacy and put forward a research idea regarding the correlation between blood drug concentration of active ingredients in TCM and clinical effectiveness. Professor Zhao contends that detecting the blood drug concentration of active ingredients in TCM can offer a crucial tool for personalized treatment in TCM clinical practice. Furthermore, he recommended employing contemporary laboratory techniques such as high-performance liquid chromatography and mass spectrometry to enhance the sensitivity and resolution of detection for active ingredients in TCM.

**Professor Guojun Zhang** (College of Laboratory Medicine, Hubei University of Traditional Chinese Medicine) introduced the team’s latest breakthroughs in the fields of clinical testing, acupuncture sensing, and Chinese medicine identification. They have developed highly sensitive diagnostic tools and explored the therapeutic mechanisms of acupuncture needles based on their conductivity. In the field of Chinese medicine, the team investigated the potential of PDVs as biomarkers. Since 2016, they have also focused on exosome detection technologies, utilizing innovative materials such as functionalized graphene to enhance detection sensitivity and specificity. These exosomes, known for their stability, are considered reliable disease biomarkers. In the study of PDVs and CHM-EVLPs, the team explored extraction and application methods, including the construction of microneedle systems to promote wound healing and antibacterial and anti-inflammatory effects. These vesicles are stable under various conditions, effectively inhibit tumor growth, and demonstrate excellent biocompatibility.

**Professor Qi Li** (Xiyuan Hospital of the China Academy of Chinese Medical Sciences) focused on research in TCM laboratory diagnostics, discussing how to leverage the advantages of laboratory departments in TCM hospitals for research purposes. He elaborated on the challenges, explorations, and future directions of TCM laboratory diagnostics. Additionally, Professor Li outlined the future research directions in this field and shared his experiences and lessons in the study of Chinese medicine and diagnostic indicators. He emphasized the importance of TCM laboratory diagnostics in solving clinical issues and how practice and advanced activities can enhance research capacity in this area.

**Professor Ximing Yang** (Dongzhimen Hospital of Beijing University of Chinese Medicine) shared insights on how laboratory medicine can contribute to the integration of Chinese and Western medicine. He first explained the concept and development of integrated Chinese and Western medicine and then demonstrated its clinical application through specific cases, such as viral infections, bacterial infections, and alcoholic liver disease. Professor Yang highlighted the role of laboratory medicine in diagnosis, treatment, and efficacy evaluation within this framework and proposed future research directions, including syndrome-based studies, preventive medicine, and Chinese medicine formula research. He also addressed the challenges faced by integrated Chinese and Western medicine in clinical and research settings, such as standardizing TCM syndromes.

**Professor Xiangdong Kang** (Shanghai University of Traditional Chinese Medicine) discussed the role of laboratory departments in the TCM diagnostic system, emphasizing their importance in disease diagnosis, treatment, and prevention. He advocated for laboratory departments to provide experimental data to support syndrome differentiation and treatment in TCM, assisting doctors in making more accurate judgments. Professor Kang also explored TCM-related research topics, including studies on TCM syndromes, pulse analysis, tongue diagnosis, TCM constitution, and Chinese medicine formulas. He highlighted the complexity of TCM syndrome research and the diagnostic variability stemming from different TCM schools and methods, as well as the diversity and intricacy of symptoms in TCM syndrome differentiation. Additionally, he emphasized the significance of Chinese medicine formula and monomer research, discussing how modern laboratory techniques can elucidate the mechanisms of Chinese medicines and optimize clinical medication regimens.

## PART 7: OPPORTUNITIES AND CHALLENGES IN THE TRANSFORMATION OF CHM-EVLPS

**Professor Hang Yin** (School of Pharmaceutical Sciences, Tsinghua University) introduced the potential applications of exosomes in the field of TCM, including their roles in the development of innovative TCM compound drugs, efficacy evaluation of new TCM drugs, and clinical research. He emphasized the importance of exosome technology in the modernization and internationalization of TCM. Furthermore, Professor Yin highlighted the advantages and immense potential of CHM-EVLPs in disease diagnosis, treatment, and drug delivery. Specifically, he pointed out that natural products, including PDVs, exhibit advantages in delivering active components and could find significant development opportunities in the health and cosmetic industries.

**Professor Lei Zheng** (Nanfang Hospital of Southern Medical University) delivered a keynote speech on new diagnostic and therapeutic technologies involving EVs. He emphasized that EVs, as new tools for disease diagnosis and treatment, represent a major breakthrough in modern medical research and may become key to advancing personalized and precision medicine. Zheng underscored Nanfang Hospital’s progress in EV detection technologies, which marks a significant step forward in research and applications in this field. Additionally, he highlighted the innovative potential of EVs in liquid biopsy and immunotherapy efficacy evaluation, particularly in single-particle precision detection, standardization, and automation of detection technologies. He called for increased international collaboration to drive the modernization and globalization of EVs technologies.

**Professor Hongbo Chen** (School of Pharmaceutical Sciences, Sun Yat-sen University) gave a keynote speech on the industrialization and large-scale production of exosomes. He emphasized the significant potential of exosomes, as key members of EVs, in biomedicine, where they may become vital tools for advancing personalized and precision medicine. Furthermore, Professor Chen highlighted the innovative potential of exosomes in drug development, particularly in large-scale preparation, quality control, and clinical application. He advocated for enhanced interdisciplinary collaboration to facilitate the modernization and industrialization of exosome technology.

## ROUNDTABLE DISCUSSION

### Topic: standardization of CHM-EVLPs: opportunities and challenges

Participants: Wei Wang, Anil K. Sood, Huangge Zhang, Chengyu Jiang, Liwen Jiang, Hua Zhou, Hang Yin, David Greening, Garine de Marcos Lousa, Zifeng Yang, Chunguang Li, and Kewei Zhao.

The experts delved into the importance and challenges of CHM-EVLPs in medical applications. They unanimously agreed that standardization is critical for ensuring the quality and efficacy of CHM-EVLPs and for promoting their acceptance and application in the global medical community. The experts stressed the need for unified naming and definitions of CHM-EVLP to facilitate effective communication and collaboration among researchers. They noted the shortcomings of PDVs compared to mammalian-derived EVs in terms of biomarker identification, which poses additional challenges for research and standardization. The experts discussed the impact of different extraction and purification methods on CHM-EVLP characteristics and emphasized the importance of establishing standardized extraction and purification processes. Furthermore, quality control was identified as a key aspect of CHM-EVLP research, with proposed standards for assessing vesicle morphology, structure, composition, and bioactivity. To better analyze and identify potential biomarkers, the experts suggested establishing a database containing CHM-EVLP research data. They also called for international cooperation to share data and resources to advance the standardization of CHM-EVLPs.

The experts expressed optimism about the clinical application potential of CHM-EVLPs and believed that standardization would facilitate their use in treating various diseases. They discussed future research directions, including deeper exploration of CHM-EVLP’s biological properties, the development of novel extraction and analysis techniques, and the fostering of international cooperation and standardization efforts. These discussions provided valuable insights and directions for the future development of CHM-EVLPs.

### Topic: clinical translation prospects of fruit and vegetable-derived EVs

Participants: Suhua Qi, Weiliang Xia, Wei Li, Hongbo Chen, Qi Lv, Junbing Fan, Jigang Wang, Chun Lin, Fahe Han.

The experts focused on the potential applications of EVLP from fruits and vegetables in cosmetics, healthcare products, and pharmaceuticals. Discussions addressed the challenges of translating EVLP into clinical applications, including ensuring efficacy, safety, standardized production, quality control, and storage. The experts emphasized the need for further research and development in these areas. They shared insights into different extraction and purification methods, highlighting the limitations of existing techniques in large-scale production. Regarding clinical translation pathways, the experts explored the potential applications of EVLP in food, health supplements, or pharmaceuticals and stressed the importance of regulatory compliance and market acceptance. The meeting also called for collaboration between researchers and industries to advance EVLP research and applications by establishing industrial alliances and platforms to facilitate information exchange and cooperation. The role of government support was highlighted, including funding and policy guidance, particularly the Guangzhou municipal government’s support for the EVLP industry chain development. The experts expressed optimism about the future prospects of EVLP in clinical translation, believing that interdisciplinary collaboration and technological innovation could overcome current challenges and enable EVLP’s widespread application in medicine and cosmetics.

### Topic: medical testing and TCM integration: innovations and breakthroughs

Participants: Junqing Tan, Weiguo Lu, Tong Wang, Qi Li, Ximing Yang, Xiangdong Kang, Kewei Zhao, Rong Su, Haibo Yu, Ming Peng.

The experts explored the role and development directions of laboratory departments in TCM hospitals in TCM research. Discussions covered how to integrate TCM characteristics with modern testing technologies to identify pioneering products with industry-leading potential and apply them in clinical settings to address specific issues. The experts emphasized the responsibility of TCM laboratory departments in improving diagnostic accuracy and advancing TCM development, as well as the challenges and opportunities in syndrome differentiation, Chinese medicine research, and other areas. They also discussed how modern methods, such as big data analysis and molecular biology technologies, could support TCM theories and improve the precision of TCM diagnostics and treatment. The participants shared their experiences and insights and expressed optimism about the future of TCM laboratory departments, believing that interdisciplinary collaboration and technological innovation could drive more breakthroughs in research and clinical applications.

### Topic: industry-academia collaboration to promote the healthy development of the exosome industry

Participants: Xu Zhang, Zhe Wang, Caihong Wu, Fang Cheng, Yunfei Chai, Lingyan Yang, Yong Xu, Shubin Xue.

The experts analyzed the development of the exosome industry and discussed how to integrate resources across the industrial chain, including equipment manufacturing, detection technologies, basic research, and capital investment, to accelerate the clinical translation of exosome technologies from the laboratory. They shared practical experiences in the extraction, purification, detection, and clinical application of exosomes and explored strategies to enhance their clinical application and industrialization. The experts also discussed technical challenges and industrialization obstacles in the field and proposed solutions through interdisciplinary collaboration and technological innovation. They expressed optimism about the industry’s future and suggested establishing an industry alliance and a public platform to enhance information exchange and cooperation, strengthen the industry’s influence, and promote the development and application of exosome technologies.

## DECLARATIONS

### Copyright

© The Author(s) 2025.

